# Detection of Thyroid Autoimmunity Markers in Euthyroid Women With Polycystic Ovary Syndrome: A Case-Control Study From Syria

**DOI:** 10.5812/ijem.17954

**Published:** 2014-07-01

**Authors:** Raghad Al-Saab, Shaden Haddad

**Affiliations:** 1 Department of Biochemistry and Microbiology, Faculty of Pharmacy, Damascus University, Damascus, Syria

**Keywords:** Anti-thyroglobulin, Anti-thyroid Peroxidase, Polycystic Ovary Syndrome, Thyroid Gland, Syria

## Abstract

**Background:**

Polycystic ovary syndrome (PCOS) is one of the most common endocrinopathies in women in reproductive age. In many cases, PCOS is associated with infertility and increased risk of miscarriage. Recent studies have detected the presence of several organ specific and nonspecific autoantibodies in women with PCOS.

**Objectives:**

The aim of this study was to evaluate the prevalence and levels of thyroid antibodies in euthyroid women with PCOS in Syria.

**Patients and Methods:**

This study included 56 euthyroid women with PCOS and 30 healthy women as a control group. PCOS was defined according to the revised 2003 Rotterdam criteria. Thyroid function was evaluated by measurement of serum TSH and FT4 levels. Antithyroid peroxidase and antithyroglobulin antibodies (anti-TPO and anti-TG, respectively) were detected as markers for thyroid autoimmunity. All parameters were measured using electrochemiluminescence immunoassay.

**Results:**

Women with PCOS had higher serum levels of anti-TPO in comparison to controls (39.9 ± 59.5 and 18.9 ± 11.2 IU/mL, respectively; P < 0.05) and no significant difference was found in serum levels of anti-TG, TSH, or FT4 between the two groups. Patients with PCOS had a higher prevalence of positive results for anti-TG and/or anti-TPO in comparison to controls (28.6% and 3.3%, respectively; P<0.05), anti-TPO alone (19.6% and 3.3%, respectively; P < 0.05) and anti-TG alone (21.4% and 3.3%, respectively; P < 0.05). No significant associations were found between antibodies and studied hormones.

**Conclusions:**

High prevalence of thyroid antibodies in euthyroid patients with PCOS refers to the importance of investigation for thyroid autoimmune state in those patients.

## 1. Background

Polycystic ovary syndrome (PCOS) is a common reproductive endocrinopathy with a reported prevalence of 3% to 15% depending on the studied population and the applied diagnostic criteria (1). It is characterized by chronic anovulatory, oligomenorrhea or amenorrhea, and signs of hyperandrogenism; in addition, it is associated with the increased rate of pregnancy loss and is considered to be the most common cause of anovulatory infertility in women in reproductive age. Despite a long history of studies on PCOS, the exact pathogenic mechanism is still unknown and it is considered as a heterogeneous disorder with both genetic and environmental components.

Autoimmune thyroid diseases (AITD) are common autoimmune disorders that affect about 5% to 20% of women in childbearing age (2). AITD is the most frequent cause of hypothyroidism in young women and it may be present without thyroid dysfunction for many years; hence, it is often ignored and results in hypothyroidism later in life (3). Many studies have reported an association between thyroid autoimmunity and adverse pregnancy outcomes including recurrent miscarriages and preterm delivery (4); moreover, recent studies have reported an association between thyroid autoimmunity and PCOS (5, 6).

For infertile women, preparation for medically assisted pregnancy comprises controlled ovarian hyperstimulation that substantially increases the circulating estrogen concentrations, which in turn can severely impair thyroid function. In women with thyroid autoimmunity, estrogen stimulation might lead to abnormal thyroid function throughout the remaining pregnancy period (7). 

Most patients with PCOS are in the child bearing age and therefore, it is important to maintain normal thyroid function before and during pregnancy to ensure the best possible outcome of the mother and progeny. 

## 2. Objectives

This study aimed to compare the prevalence and levels of thyroid autoantibodies in a group of Syrian euthyroid women with PCOS and a control group of women in reproductive age to determine whether women with PCOS were at a greater risk of thyroid autoimmune diseases or thyroid dysfunction. 

## 3. Patients and Methods

### 3.1. Study Participants

This case-control study was performed between January and December 2012 in Damascus, Syria. Women with signs of hyperandrogenism and/or oligomenorrhea visiting obstetrics and gynecology clinics were included in our study. PCOS was defined by credentialed gynecologists according to the revised 2003 Rotterdam criteria (8), which requires the presence of at least two of the three following indicators:

- ovulatory disturbance, mainly oligomenorrhea or amenorrhea;
- hyperandrogenism as defined either clinically by hirsutism, or severe acne/seborrhea, and/or biologically by elevated levels of total or free testosterone;
- and polycystic ovaries at ultrasonography (9).

Controls were females in reproductive age with regular menstrual cycles, no signs of hyperandrogenism, normal ovaries on pelvic ultrasound examination, and normal serum levels of free testosterone. 

The total number of participants at the beginning of study was 119. We excluded the medical conditions that cause irregular menstrual cycles and androgen excess such as hyperprolactinemia (three women), hypothyroidism (five women), and hyperthyroidism (one woman); we also excluded women who were taking oral contraceptives or corticosteroids (nine women) as well as patients who did not fulfil Rotterdam criteria (six women). In order to include only euthyroid subjects, women with abnormal thyroid stimulating hormone (TSH) levels (nine women) were also excluded from the study. 

A total of 56 euthyroid women with PCOS were included in the study group, whereas 30 euthyroid normally ovulating women were studied as a control group.

A detail history was taken that included current age, age at menarche, history of menstrual irregularity, acne, hirsutism, infertility, obstetric history, thyroid disorders, history of similar disorders in the family, contraceptive methods, and the current medications. All participants recruited voluntarily in the study and signed the informed consent form.

### 3.2. Measurement of Laboratory Parameters

Blood samples were collected using clot activator tube in the morning between the second and the fifth day of menstrual cycle. Serum was separated after a standardized time and was kept frozen at -80℃ for further analysis.

The following serum measurements were performed: TSH (standard reference range, 0.27-4.2 µIU/mL; intra-assay coefficient of variation [CV], 1.5%-8.6%; and inter-assay CV, 1.8%-8.7%), free thyroxine (FT4; reference range 0.93-1.7 ng/dL; intra-assay CV, 1.4%-2.9%;and inter-assay CV, 2.7%-6.6%), antithyroid peroxidase antibody (anti-TPO;reference value < 34 IU/mL; intra-assay CV, 2.5%-7.0%; and inter-assay CV, 7.1%-24.4%), and antithyroglobulin antibody (anti-TG; reference value < 115 IU/mL; intra-assay CV, 4.6%-5.6%; and inter-assay CV, 5.9%-8.7%). All measurements were performed using electrochemiluminescence kits (Roche diagnostics GmbH, Mannheim, Germany) and estimated on Elecsys 2010 (Roche diagnostics, Indianapolis, IN). Assay reliability was determined by the use of commercially derived control sera of low and high concentrations.

### 3.3. Statistical Analysis

Values were reported as mean ± standard deviation (SD). Mean values were compared using Student's t test and differences in positive results between groups were tested using Chi square test. The association of both anti-TPO and anti-TG with TSH and FT4 were assessed using Pearson’s correlation test. A P value < 0.05 was considered statistically significant. All statistical analysis was done by using IBM SPSS version 20 (SPSS Inc, Chicago, IL, USA).

## 4. Results

The mean age of the patients with PCOS and controls was 23.8 ± 5.6 years (range, 15-34) and 28.9 ± 5.8 years (range, 16-39), respectively. Five patients (8.9%) had family history of hypothyroidism, hyperthyroidism, or goiter among which two patients had positive results for anti-TPO and anti-TG; one had just positive results for anti-TG and personal history of goiter while one of the two patients who had negative results for antibodies had personal history of goiter. One patient (1.8%) who had positive results for anti-TG had an uncle and sisters with thyroid autoimmunity. Amongst controls, only one woman (3.3%) who had negative results for antibodies had a family history of goiter. Table 1 compares the intervening variables between patients with controls.

At time of collecting samples none of the participants had mentioned the administration of iodine supplements; however, all of them were using iodinated salt in the diet.

Our results showed that positive results for anti-TG and anti-TPO were more prevalent in women with PCOS and serum levels of anti-TPO were significantly higher in those women in contrast to the controls; however, serum levels of anti-TG, TSH, and FT4 of the patients with PCOS and the controls did not differ significantly. Table 2 shows the biochemical characteristics of patients and controls.

From 56 euthyroid patients with PCOS, 11 (19.6%) patients had positive results for anti-TPO, in comparison to only one woman (3.3%) in the control group (χ^2^ = 4.3, P = 0.037). Positive results for anti-TG was seen in 12 (21.4%) patients and 1 woman (3.3%) in the control group (χ^2 ^= 4.9, P = 0.026). 

**Figure 1.**
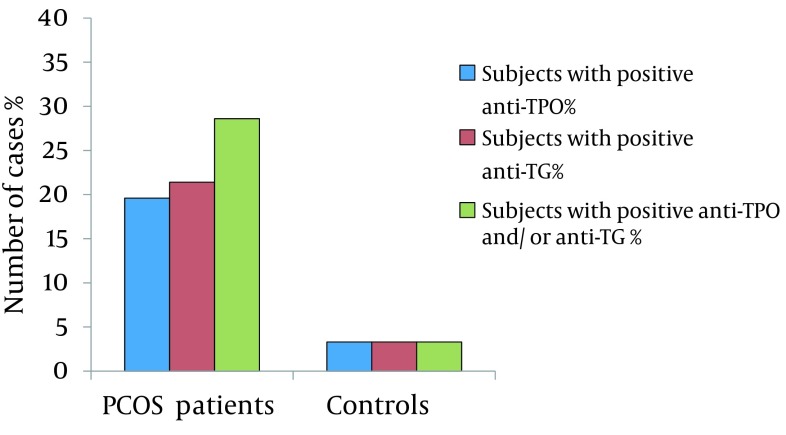
Prevalence of Antithyroid Peroxidase and Antithyroglobulin Antibodies Positivity in Patients with Polycystic Ovary Syndrome and Controls

Positive results for either anti-TPO or anti-TG or both was noticed in 16 (28.6%) patients and only 1 (3.3%) control (χ^2^ = 7.8, P = 0.005). Figure 1 shows the prevalence of the positive results for anti-TPO and anti-TG in patients and controls.

The TSH serum levels were less than 2.5 in 37 patients of which eleven (29.7%) had positive results for anti-TPO and/or anti-TG.

No significant correlation was revealed between anti-TPO and TSH (r = 0.15, P = 0.25) or between anti-TPO and FT4 (r = -0.006, P = 0.96) in patients. No significant correlation was revealed between anti-TG and TSH (r = -0.16, P = 0.23) or between anti-TPO and FT4 (r = -0.1, P = 0.45) in patients.

**Table 1.** Comparison of the Variables Between Patients and Controls ^a,b^

Variable	Controls	PCOS
**Age, y**	28.9 ± 5.8	23.8 ± 5.6
**BMI, kg/m** ^**2**^	23.9 ± 2.9	24.9±5.9
**Family History of Hypo/Hyperthyroidismor Goiter**	3.3%	8.9%
**Family History of** **Thyroid Autoimmunity**	-	1.8%
**Personal History For Hypo/Hyperthyroidism or Goiter**	3.3%	3.6%
**Diabetes Mellitus**	-	1.8%
**Smoking**	-	7.1%

^a^ Abbreviations: BMI, body mass index; PCOS, polycystic ovary syndrome.

^b^ data are presented as mean ± SD or frequency.

**Table 2.** Comparison of Biochemical Characteristics Between Patients and Controls ^a,b^

Variable	Controls (n = 30)	PCOS (n = 56)	P Value
**TSH, µIU/mL**	2.1 ± 0.85	2.11 ± 0.96	0.95
**FT4, ng/dL**	1.18 ± 0.14	1.24 ± 0.15	0.059
**Anti-TPO, IU/mL**	18.9 ± 11.2	39.9 ± 59.5	0.013
**Anti-TG, IU/mL**	35.5 ± 62.6	102.5 ± 243	0.056

^a^ Abbreviations: PCOS, polycystic ovary syndrome; TSH, thyroidstimulating hormone; FT4, free thyroxine; Anti-TPO, antithyroid peroxidase antibody; and Anti-TG, antithyroglobulin antibody

^b^ Data are presented as mean ± SD

## 5. Discussion

In the present study, significantly higher prevalence of thyroid autoantibodies in patients with PCOS in comparison to the controls was noted. Out of 56 euthyroid patients with PCOS, 16 (28.6%) had positive results for anti-TG and/or anti-TPO in comparison to the controls with only one (3.3%) positive result in 30 participants. The exact prevalence of PCOS in Syrian population is unknown; however, based on the diagnosis rate of new cases, it has become obvious that PCOS is a common cause of pregnancy complications among Syrian women in reproductive age.

Patients with PCOS often have defective progesterone secretion which leads to an increased estrogen to progesterone ratio. Estrogen can increase the expression of interleukin-6 in T cells and the absence of inhibitory action of progesterone may lead to overstimulated immune system and makes these patients more prone to autoimmune disorders (10). Increased levels of circulating C-reactive protein (CRP) has been demonstrated in patients with PCOS, which is considered a kind of low-grade inflammation (11).

Recently, several researchers showed that some serologic markers of autoimmunity were elevated in patients with PCOS, and various systemic and organ-specific autoantibodies have been recognized in patients with PCOS (12). Thyroid hormones have an important role before and during pregnancy and to our knowledge, there was no study that had evaluated the thyroid autoimmunity in patients with PCOS in Syria; hence, it seemed necessary to conduct a study concerning this syndrome.

These findings are very close to the findings of Janssen et al. (5) study in Germany where elevated levels of anti-TPO or anti-TG were found in 47 (26.9%) of 175 patients with PCOS in comparison to only 14 (8.3%) out of 168 controls. A study from Turkey found that 37.8% of 107 patients with PCOS had positive anti-TPO or anti-TG (13), While another study from India reported positive results for anti-TPO in 22.5% of 80 patients with PCOS in contrast to 1.25% positive results of 80 controls (14). The small differences in the percentage between studies could be attributed to different value of cut point and the size of studied groups. Our study is the first one that was conducted in euthyroid patients with PCOS and this gives importance to the investigation of thyroid autoimmunity in this group of patients. Patients with anti-TPO and anti-TG are more likely to develop thyroid dysfunction later in life.

In its recent guidelines, the National Academy of Clinical Biochemistry (NACB) recommended the use of 2.5 µIU/mL, rather than 4 µIU/mL for TSH levels, due to the fact that the populations in which the definition of the reference range is based, include persons undergoing an initial phase of autoimmune thyroid disease (15). Of 37 patients with PCOS that have TSH levels below 2.5 µIU/mL, 11 (29.7%) patients had positive results for anti-TPO and/or anti-TG. The percentage of positive results did not differ after exclusion of patients with PCOS and TSH levels above 2.5 µIU/mL and it was very close to other studies like the study of Janssen et al. (5) where patients with autoimmune thyroiditis were included. It means that, irrespective of thyroid function, patients with PCOS have a higher prevalence of thyroid autoantibodies.

The mean serum anti-TPO was significantly higher in patients with PCOS in comparison with women in the control group (P = 0.013), whereas serum anti-TG levels were higher in patients than in controls; however, the difference between the two groups was not statistically significant. Among the most recently studies, Kachuei et al. (6) from Iran has also shown significantly higher levels of serum anti-TPO in patients with PCOS than in controls (216 ± 428 vs. 131 ± 364 IU/mL; P = 0.04); however, serum levels of anti-TG did not show any difference between groups.

In this study, TSH and FT4 levels did not differ significantly between patients with PCOS and controls while some studies such as Janssen et al (5). reported that TSH levels were higher in patients with PCOS; this may be explained by considering that we only included euthyroid participants in our study. These findings require more investigations to understand the underlying association between PCOS and thyroid autoimmunity and the mechanism by which this common condition alters pregnancy outcomes.

### 5.1. Limitations

Data concerning family and personal medical history were obtained by asking participants while they were filling data sheet, and we could not obtain data from medical files. Although clinical investigation of goiter and thyroid ultrasound would have been valuable, we were unable to provide it because of financial limitations.

### 5.2. Conclusions

The present findings show that PCOS was associated with increased positive results for thyroid autoantibodies in our population; in addition, the patients with PCOS had an increased risk of thyroid disorders. Serum autoantibodies against thyroid should be searched in patients with PCOS who decide to get pregnant even when there is no evidence of overt thyroid dysfunction.
